# Alexithymia, self-reported external gain expectations, and overreporting on symptom validity tests in hospital patients: further evidence for the relevance of alexithymia

**DOI:** 10.1093/arclin/acag048

**Published:** 2026-07-10

**Authors:** Brechje Dandachi-FitzGerald, Sabine Hoes, Rudolf Ponds, Harald Merckelbach

**Affiliations:** Faculty of Psychology, Open University, Heerlen, The Netherlands; Faculty of Psychology and Neuroscience, Maastricht University, Maastricht, The Netherlands; Faculty of Psychology and Neuroscience, Maastricht University, Maastricht, The Netherlands; Department of Medical Psychology, Amsterdam University Medical Center, Amsterdam, the Netherlands; Faculty of Psychology and Neuroscience, Maastricht University, Maastricht, The Netherlands

**Keywords:** symptom validity, symptom overreporting, alexithymia, external gain, malingering, hospital patients

## Abstract

**Objective:**

Symptom overreporting is often considered to be moderated by external incentives, such as financial or legal advantages, although other factors may also play a role. Preliminary studies have suggested a connection between symptom overreporting and alexithymia, that is, trait-like difficulties in recognizing and describing internal sensations. This study aimed to further clarify the relationships among external gain expectations, alexithymia, and symptom overreporting. Specifically, we examined whether alexithymia is related to overreporting in patients without self-reported external gain expectations.

**Method:**

Using a cross-sectional design, patients referred for psychological assessments in a hospital setting completed a questionnaire about external gain expectations (e.g., regarding work, housing, legal issues). We differentiated between those with self-reports of external gain expectations (*n* = 73) and those without (*n* = 84). Both subsamples were administered the Toronto Alexithymia Scale-20 (TAS-20), the Structured Inventory of Malingered Symptomatology (SIMS), and the Minnesota Multiphasic Personality Inventory-2 Restructured Form (MMPI-2-RF).

**Results:**

Across the full sample, alexithymia showed a positive and statistically significant association with symptom overreporting on the SIMS and the Infrequent somatic responses scale (Fs) of the MMPI-2-RF: *r* = 0.44 and *r* = 0.31, respectively. These positive associations were also evident in the subgroup without self-reported external gain expectations (i.e., *r* = 0.35, 95% CI [0.14, 0.52] and *r* = 0.35, 95% CI [0.15, 0.53], respectively). Regression analysis indicated that self-reported external gain expectations did not account for the relationship between symptom overreporting and alexithymia.

**Conclusion:**

These findings suggest that alexithymia is associated with symptom overreporting independently of self-reported external gain expectations. More broadly, the results raise the possibility that alexithymic traits may compromise the accuracy of symptom reporting itself. If so, this has implications not only for the interpretation of symptom validity tests, but also for the broader use of self-report measures in clinical assessment.

## Introduction

Some patients may overexpress their complaints and limitations, a behavior that can be detected using validity tests. Such tests are categorized into two types: symptom validity tests (SVTs), detecting exaggerated self-reports, and performance validity tests (PVTs), identifying invalid cognitive performance (Larrabee, 2012). In the current article, we will primarily focus on SVTs such as the Structured Inventory of Malingered Symptomatology (SIMS; Smith & Burger, 1997; Shura et al., 2022; van Impelen et al., 2014) and the overreporting indicators of the Minnesota Multiphasic Personality Inventory-2-RF (MMPI-2-RF; e.g., Aparcero et al., 2023). These tools are often used in both clinical and forensic assessments (Martin et al., 2024) and typically include items targeting rare or nonexistent symptoms, with excessive endorsement indicating of symptom overreporting.

What motivates patients to overexpress their symptoms on SVTs? One commonly voiced view, particularly in the forensic context, is that symptom overreporting may be driven by expectations of external gain. In that context, such expectations are prevalent, and their relevance is reflected in Criterion A of widely used malingering guidelines (Sherman et al., 2020). For certain groups of patients, such as those involved in independent medicolegal assessments related to litigation or insurance procedures, there is robust evidence to support the intimate link between symptom overreporting and external gain expectations. For instance, Bianchini et al. (2006) examined patients with work-related traumatic brain injuries (*N* = 332) and found that the extent of financial compensation, rather than the severity of the brain injury, was associated with overexpression of complaints and limitations as assessed by SVTs and PVTs. This led the authors to conclude that “[C]ombined with previous research, this study reinforces the notion that financial incentive motivates intentional symptom exaggeration” (Bianchini et al., 2006, p. 844).

In non-forensic settings, external incentives may also engender symptom overexpression (e.g., Schroeder et al., 2022), but their role appears to be less pronounced (e.g., Merckelbach et al., 2025). For instance, Dandachi-FitzGerald et al. (2016) examined hospital outpatients (*N* = 469) referred for psychological assessment and found that, depending on diagnostic category, between 12% and 19% scored above the SIMS cutoff. The authors asked clinicians familiar with the patients and their backgrounds to complete a checklist identifying potential external gain motivations, such as involvement in legal proceedings or receipt of disability payments. These motivations emerged as weak predictors of symptom overreporting, making it unlikely that they could fully explain elevated SVT scores (see also Martin & Schroeder, 2020).

Experts in the field (e.g., Sweet et al., 2021) have long recognized that, beyond the anticipation of external incentives, deviant scores on SVTs and/or PVTs may arise from a variety of other factors. However, our understanding of these factors remains limited. Focusing specifically on PVTs, Schroeder and Martin (2022, pp. 25–26) reviewed the extant literature and concluded as follows: “The empirical research also indicates that many previously proposed explanations of PVT failure (i.e., apathy, fatigue/daytime sleepiness, pain, medication effects, depression or anxiety, a “cry for help”) do not actually cause PVT failure in the vast majority of cases, although rare exceptions might potentially occur when there are extreme presentations.”

In our view, much the same holds for deviant scores on SVTs. Although symptom overreporting in clinical settings has often been attributed to personality traits, the overall evidence for such association remains weak (Van Helvoort et al., 2022). One potential exception is alexithymia. People with this trait experience chronic difficulties in identifying and labeling bodily sensations (Bagby et al., 2020, 2021b). Research summarized by Luminet and Nielson (2025) converges on the notion that alexithymia might manifest as overresponding. For example, in their memory study, Meltzer and Nielson (2010) observed that compared with controls, high alexithymic individuals recalled illness-related words significantly more often than negative words. The authors speculated that this memory bias may reflect somatosensory amplification, that is, a proclivity to experience symptoms as intense and threatening. Likewise, De Gucht and Heiser (2003) concluded in their review that alexithymia is related to stronger symptom expression, whereas several other studies indicated that this stronger symptom expression is independent of the presence of somatic disease conditions (e.g., Lumley et al., 2007).

So far, only two studies examined whether alexithymia is associated with elevated scores on SVTs. In a pioneering study, Brady et al. (2017) administered the Toronto Alexithymia Scale (TAS-20; Bagby et al., 1994) and the Miller Forensic Assessment of Symptoms Test (M-FAST; Miller, 2001; Detullio et al., 2019) to veterans with PTSD (*N* = 75). The authors observed a significant and positive correlation (*r* = 0.49) between TAS-20 and M-FAST. They suggested that “[A]lexithymia should be considered as a potential mechanism contributing to the overreporting phenomena observed in the assessment and treatment of PTSD, and warrants further study” (Brady et al., 2017; p. 80).

Merckelbach et al. (2018) investigated the relationship between the TAS-20 and overreporting on the SIMS in forensic psychiatric outpatients (*n* = 40) and non-forensic participants recruited from local football clubs (*n* = 40). Consistent with Brady et al.’s (2017) findings, a significant positive association between alexithymia and symptom overreporting was evident for both the forensic and non-forensic subsample (*r* = 0.56 and *r* = 0.51, respectively).

The studies by Brady et al. (2017) and Merckelbach et al. (2018) did not directly examine whether external gain motivations may foster overreporting that spills over into self-report measures generally, including the TAS-20. However, the observed correlational pattern between symptom overreporting and the TAS-20 subscales (Bagby et al., 2020; Luminet & Nielson, 2025)—difficulty identifying feelings (DIF), difficulty describing feelings (DDF), and an externally oriented thinking style (EOT)—indirectly argues against this interpretation. If external gain would engender a diffuse overreporting tendency, one would expect indiscriminate positive correlations across all three subscales. Yet, both Brady et al. (2017) and Merckelbach et al. (2018) consistently observed that the correlations between DIF and overreporting were highest (*r* = 0.60 and *rs* = 0.57 and 0.58, respectively), whereas those between EOT and overreporting were lowest (*r* = 0.14 and *rs* = 0.02 and 0.22, respectively). However, neither study explicitly examined the role of external gain expectations.

Differentiating between a group with and a group without external gain motivations heavily relies on the patients' willingness to disclose their expectations. Van Egmond and Kummeling (2002) explored this by using a checklist to inquire about desirable incentives outside the scope of treatment, such as employment, social security, or housing, among psychiatric outpatients (*N* = 166). The authors found that patients were often surprisingly forthcoming about these motives. In fact, a sizeable minority of patients (42%) in their study reported to expect external gain as part of their treatment.

In this study, we aimed to further clarify the relationships between external gain, alexithymia, and symptom overreporting. Specifically, we distinguished between two groups of hospital patients referred for psychological problems: one group expressed a desire to pursue specific incentives and expected clinicians' assistance in achieving them (i.e., external gain motivations), while the other group reported no such incentive-driven expectations (i.e., no external gain motivations). If raised alexithymia scores were an overreporting artifact of external gain motivations, one would expect a significant correlation between alexithymia and symptom overreporting in the first, but not in the second group. However, if alexithymia is independently associated with symptom overreporting, one would expect positive and significant correlations between the two variables in both groups.

## Methods

### Study design

This study used a consecutive sampling method and a between-subjects design with two groups: patients with one or more self-reported secondary gain expectations and patients who did not report external gain expectations. The hypotheses and analysis plan were preregistered on the Open Science Framework after data collection but prior to data analysis (link: https://osf.io/g34y9/registrations).

### Patients

The study used data from routine psychological assessments in Maastricht University Medical Centre+ (MUMC+). The study protocol was reviewed by the Medical Ethics Committee of MUMC+ (METC 2017–0278) and was deemed not to be subject to the Dutch Medical Research Involving Human Subjects Act (WMO). The sample included hospital patients referred for assessments between March 2018 and February 2019. Assessments were conducted only when patients could participate meaningfully; accordingly, patients in an acute psychotic state, with severe cognitive impairments, or uncorrectable sensory deficits were excluded. During the data collection period, 331 consecutive psychological assessments met clinical feasibility criteria. For this study, we included only assessments that used the department’s standard psychological assessment battery, comprising the TAS-20, SIMS, BSI, and MMPI-2-RF, and excluded assessments based on specialized test batteries without these core measures. This resulted in the exclusion of 115 assessments, most notably evaluations related to eating disorders (*n* = 44), neuropsychological assessments (*n* = 24), and psychodiagnostic evaluations of sexual dysfunctions (*n* = 4). In addition, repeated assessments (*n* = 11) were excluded, as only first-time psychological assessments were considered eligible. Assessments were also excluded when indications of intellectual disability emerged during the assessment (*n* = 7), as this may compromise the validity of self-report questionnaire data. Additional assessments were excluded because of missing background data (*n* = 8), insufficient Dutch proficiency (*n* = 7), discontinued assessments (*n* = 3), no-shows (*n* = 3), and other reasons (*n* = 4). This left 216 eligible assessments. Within this group, 40 assessments (19%) were excluded because of missing project-relevant measures (TAS-20, SIMS, or BSI), whereas 8 (5%) were omitted due to inattentive responding on the MMPI-2-RF (VRIN or TRIN T-score ≥ 80).

The final sample consisted of 168 patients (80 men), with a mean age of 44.9 years (*SD* = 14.9, range: 18–83 years). In terms of the International Standard of Education (UNESCO Institute for Statistics, 2012), 9.5% had a lower education, 49% a medium education, and 42% a higher education. Most psychological assessments were conducted at the hospital’s outpatient Psychology and Psychiatry department (*n* = 83; 48.2%), followed by assessments of patients admitted to its psychiatric ward (*n* = 73; 43.5%). A smaller proportion (*n* = 14; 8.3%) took place at the hospital’s MedPsych Unit, a specialized service for patients with unexplained somatic and psychological symptoms. Patients fell into the following diagnostic categories: mood disorders (*n* = 83; 49%), somatic symptom disorders (*n* = 34; 20%), anxiety disorders (*n* = 18; 11%), personality disorders (*n* = 10; 6%), psychotic disorders (*n* = 10; 6%), and miscellaneous diagnoses (*n* = 7; 4%). Diagnostic information was missing in six cases (4%)*.*

Patients completed an external gain questionnaire, a 12-item checklist based on the work of Van Egmond and Kummeling (2002). The checklist asks whether patients expect clinicians to assist them in obtaining support in areas such as employment, housing, financial matters, and insurance claims. The checklist has no established normative data and no cutoff score. Specifically, patients were first asked to rate the strength of their expectations on a 9-point scale (1 = *not at all strong*; 9 = *extremely strong*). Following this, they received a list of 12 specific domains, each with a checkbox, and were asked to tick the boxes for the domains in which they expected clinicians to assist them. Overall, 73 patients (43.5%) indicated that they had external gain expectations in one or more domains (see Table 1), with a median number of two domains (IQR = 1–3.5). The remaining 84 patients (50%) reported no external gain expectations. Eleven patients (6.5%) did not complete the external gain questionnaire.

**Table 1:** Frequency of domains in which patients indicated they expected the clinician could help them. Data presented for the subsample of patients who reported one or more external gain expectations (*n* = 73)

**External gain domains**	***n***	**%**
Work	46	63.0
Study/training	35	47.9
Housing	20	27.4
Sick leave	16	21.9
Reimbursement for exceptional medical expenses (e.g., plastic surgery)	12	16.4
Financial problems	12	16.4
Assistance with care of a family member (e.g., household help, family support, hospitalization of partner/family member)	11	15.1
Social security benefits	9	12.3
Permission to work from home	8	11.0
Legal/police matters	8	11.0
Insurance	7	9.6
Extended vacation	4	5.5

### Measures

#### Toronto Alexithymia Scale-20

The TAS-20 (Bagby et al., 1994, 2020) is a self-report questionnaire consisting of 20 items that assess three key dimensions of alexithymia: DIF, DDF, and EOT. Each item is rated on a 5-point Likert scale ranging from 1 (*strongly disagree*) to 5 (*strongly agree*), with total scores ranging from 20 to 100. Elevated scores reflect higher levels of alexithymia, with scores above 60 considered clinically significant. Research summarized by Bagby et al. (2020; 2021a) and Luminet and Nielson (2025) shows that the TAS-20 possesses good psychometric properties, including adequate convergent and divergent validity, as well as a stable three-factor structure across multiple languages and in clinical as well as in non-clinical samples. Furthermore, clinician-administered interviews to assess alexithymia generally align well with self-reported alexithymia measured by the TAS-20.

We used the Dutch version of the TAS-20 (Kooiman et al., 2002). Internal reliability for the full TAS-20 scale was 0.815, whereas reliabilities for DIF, DDF, and EOT were 0.798, 0.795, and 0.451, respectively. One patient had a missing score on one of the TAS-20 items. In this case, the missing value was imputed based on the patient's average score on the remaining items.

#### Structured Inventory of Malingered Symptomatology

The SIMS (Smith & Burger, 1997) is a 75-item SVT designed to detect symptom overreporting, using a yes/no response format. Its items describe rare, atypical, or extreme cognitive or psychopathological symptoms that genuine patients are unlikely to endorse. The more items patients endorse, the higher the likelihood they are engaging in overreporting. Reviews by van Impelen et al. (2014) and Shura et al. (2022) suggest that while the SIMS demonstrates good sensitivity at lower cutoffs (i.e., <16), its specificity remains suboptimal (see also Wisdom et al., 2010). Dandachi-FitzGerald et al. (2020a) administered the Dutch version of the SIMS to neurological patients, including those with conditions such as dementia or Parkinson's disease (*N* = 120). A minority, 12%, scored above the cutoff of >16. Based on these considerations, we adopted a cutpoint of 16 for the Dutch version of the SIMS, while also conducting a follow-up analysis using the more conservative threshold of 19. In the present study, the full SIMS scale demonstrated good internal reliability (α = 0.833).

#### Minnesota Multiphasic Personality Inventory-2- Restructured Form

Several authors have noted the overlap between the SIMS and the standard validity scales of the MMPI-2-RF (Ben-Porath & Tellegen, 2008/2011), such as the F-r (infrequent responses scale) and Fp-R (infrequent psychopathology responses scale), and the incremental validity of using these measures together is not yet well established (Shura et al., 2022). However, one interesting exception is Fs (Infrequent somatic responses scale; for example, Sellbom et al., 2012), which is particularly relevant in medical settings. The Fs, consisting of 16 items, was specifically developed for the MMPI-2-RF to capture somatic complaints that are rarely endorsed by hospital patients. Consequently, elevations on this scale are likely to reflect overreporting of somatic symptoms in hospital patient samples. In the current study, we focused on Fs of the Dutch version of the MMPI-2-RF. Following the review of Sleep et al. (2015), we used >100 T as the primary cutoff, while also examining elevations above 80 T. Sánchez et al. (2017) found in their patient group (*N* = 308) the internal reliability of the Fs to be 0.709.

#### Brief Symptom Inventory

The BSI is a 53-item tool used to assess self-reported psychological symptoms in various areas, including depression, anxiety, and psychoticism (Derogatis, 1975). Participants indicate the extent to which each symptom affected them on a 5-point Likert scale, ranging from 0 (*not at all*) to 4 (*extremely*). In the present study, we utilized the Dutch version of the BSI, known for its excellent psychometric properties (De Beurs, 2011). Our focus was on the Global Severity Index (GSI), which represents the average score across all areas. A widely used cutoff for this index is >0.50, which is associated with a sensitivity of 0.84 and a specificity of 0.70 (De Beurs & Zitman, 2006). The internal consistency for the full scale in this study was 0.953.

### Procedure

Patients referred to the hospital's psychology department completed a tailored test battery. For this study, only those with complete data for the TAS-20, SIMS, MMPI-2-RF, and BSI were included. Psychological testing was administered by certified psychological assistants under clinical psychologist supervision. Assistants were blind as to the hypotheses that we addressed in our research project. Assessment duration averaged roughly two hours, varying by patient and battery. The order of the tests differed across patients. All measures and instructions were in Dutch.

### Data analysis

We analyzed the data in three stages. In the first stage, we obtained descriptive statistics for the entire sample (*N* = 168) and computed Pearson correlations between alexithymia (TAS-20), symptom overreporting indices (SIMS and Fs), and Global Severity Index (GSI) scores of the BSI. We also examined the Pearson correlations between TAS-20 subscales and overreporting indices, using Bonferroni correction (0.05/6 = 0.008).

In the second stage, we compared correlations between alexithymia and symptom overreporting in patients with (*n* = 73) and without (*n* = 84) self-reported external gain expectations. Patients with incomplete data on the external gain questionnaire were excluded. Group differences in correlations were assessed using 95% confidence intervals, and Fisher's exact tests were conducted to compare proportions of patients exceeding cutoffs for alexithymia and symptom overreporting measures.

In the third stage, we conducted a regression analysis with self-reported external gain and alexithymia as predictors of a compound variable of symptom overreporting, consisting of the average of *Z*-transformed SIMS and Fs T scores. The GSI was not included as a covariate. Our aim was not to determine whether alexithymia predicts SVT scores independently of all forms of psychopathology or distress, but rather to examine whether, in a clinically distressed population, alexithymic characteristics are associated with symptom overreporting independently of self-reported external gain expectations. From that perspective, including GSI as a covariate risks overcorrection and may obscure clinically meaningful associations.

## Results

### Correlations between alexithymia and symptom (over)reporting in full sample

As shown in Table 2, alexithymia correlated positively with symptom overreporting measures and the GSI (all *p*s < .001). Examining the subscales of the TAS-20 and overreporting measures corroborated earlier findings by Brady et al. (2017) and Merckelbach et al. (2018). Specifically, DIF exhibited significant correlations with both the SIMS (*r* = 0.46, *p* <.001) and Fs (*r* = 0.39, *p* <.001). DDF correlated significantly with the SIMS (*r* = 0.30, *p* <.001) but not with Fs when applying Bonferroni corrections (*r* = 0.17, *p* = .032). Correlations between EOT and overreporting indices were not significant (SIMS: *r* = 0.18, *p* = .021; Fs: *r* = 0.08, *p* = .283). Most patients scored above the GSI cutoff, underscoring the clinical status of our sample.

**Table 2:** Descriptive statistics and Pearson-product moment correlations between tests in the full sample (*N* = 168)

	*M* (*SD*)	*n* (%) > cutpoint	SIMS	Fs	GSI
TAS-20	54.88 (11.25)	54 (32.1%)	0.44^a^	0.31^a^	0.50^a^
SIMS	12.53 (6.54)	44 (26.2%)		0.54^a^	0.61^a^
Fs	65.71 (17.41)	6 (3.6%)			0.37^a^
GSI	1.17 (0.67)	137 (81.5%)			

^a^ *p* < .001

*Note*: TAS-20 = 20-item Toronto Alexithymia Scale; SIMS = Structured Inventory of Malingered Symptomatology; Fs = Infrequent somatic responses scale of the Minnesota Multiphasic Personality Inventory–2 Restructured Form (MMPI-2-RF); GSI = Global Severity Index of the Brief Symptom Inventory.

### Comparisons between groups with and without self-reported external gain expectations

Table 3 shows Pearson correlations between alexithymia and overreporting indices in groups with and that without self-reported external gain expectations, as well as the proportions of patients exceeding the cutoffs on the various measures.

**Table 3:** Pearson product–moment correlations (and their 95% CI’s) between TAS-20, SIMS, and Fs of the MMPI-2-RF in patients with (*n* = 73) and without (*n* = 84) self-reported external gain expectations. Numbers (proportions) in both groups who scored above the cutoffs of TAS-20, SIMS, and Fs are also shown

	Self-reported external gain expectations			
	Yes (*n* = 73)	No (*n* = 84)	*Z/* ꭓ2	*p*
*r*(TAS-20, SIMS)	0.54 [0.34, 0.68]	0.35 [0.14, 0.52]	1.46	.072
*r*(TAS-20, Fs)	0.27 [−0.04, 0.47]	0.35 [0.15, 0.53]	0.54	.294
*n* (%) > cutpoint TAS-20 (60)	22 (30.1%)	29 (34.5%)	0.59	.555
*n* (%) > cutpoint SIMS (16)	19 (26.0%)	24 (28.6%)	0.36	.719
*n* (%) > cutpoint Fs (100 T)	3 (4.1%)	3 (3.6%)	0.18	.857

*Note:* TAS-20 = 20-item Toronto Alexithymia Scale; SIMS=Structured Inventory of Malingered Symptomatology; Fs = Infrequent somatic responses scale of the Minnesota Multiphasic Personality Inventory–2 Restructured Form (MMPI-2-RF). ꭓ2 test statistics and their corresponding *p*-values for comparisons involving proportions are also shown.

Three remarks are in order. First, as the 95% confidence intervals show, the significant alexithymia-overreporting link is evident in both groups and the groups do not differ in the strength of these links. This is also evident when correlations in both groups are compared using the *z*-statistic: no significant differences between groups were found in the associations between TAS-20 and SIMS or between TAS-20 and Fs.

Second, the groups with and without self-reported external gain incentives do not differ significantly in the proportions of individuals exceeding the TAS-20, SIMS, or Fs cutpoints, with Fisher's exact test *p*-values of .508, .720, and .704, respectively. Table 3 gives the Chi-square values for these comparisons.

Third, given the skewed distribution of measures (e.g., the SIMS), we reran the analyses using Spearman rank correlations. Additionally, we applied a stricter SIMS cutoff of 19, following van Impelen et al. (2014). We also considered the more liberal Fs cutoff of 80 T. The results showed a consistent pattern: there were no group differences in the correlations between TAS-20 and SIMS among participants with versus without self-reported gain expectations. The absence of group differences also persisted when more stringent SIMS and Fs cutoffs were applied together with Bonferroni corrections (Supplementary Table 1).

### Regression analysis with self-reported external gain incentives and alexithymia as predictors

Table 4 summarizes the results of the regression analysis, in which alexithymia and self-reported external gain served as predictors of a composite symptom overreporting variable, derived from averaging z-transformed SIMS scores and T-transformed Fs scores. External gain was treated as a dichotomous variable (no self-reported external gain expectations vs. one or more). In the third and final step, we added the interaction term between alexithymia and self-reported external gain. Overall, the regression analysis showed that alexithymia accounted for variance in symptom overreporting, whereas self-reported external gain expectations did not explain additional variance beyond alexithymia in this sample of hospital patients.

**Table 4:** Regression analysis predicting compound overreporting with self-reported external gain expectations (SREGE) and alexithymia as predictors

**Variable**	**B**	**95% CI for B**		**SE B**	**β**	**R** ^**2**^	**Δ R** ^**2**^
		**LL**	**UL**				
Step 1						0.11	0.11^a^
Constant	18.71	12.02	25.41	3.39			
TAS-20	0.26^a^	0.14	0.38	0.06	0.33^a^		
Step 2						0.11	0.003
Constant	19.11	12.31	25.92	3.45			
TAS-20	0.26^a^	0.14	0.38	0.06	0.33^a^		
SREGE(yes/no)	−0.92	−3.61	1.77	1.36	−0.05		
Step 3						0.12	0.000
Constant	18.30	9.45	27.15	4.48			
TAS-20	0.28^a^	0.12	0.43	0.08	0.35^a^		
SREGE (yes/no)	1.00	−12.62	14.63	6.90	0.06		
TAS-20× SREGE (yes/no)	−0.04	−0.28	0.21	0.12	−0.11		

*Note: N* = 157; TAS-20 = 20-item Toronto Alexithymia Scale; SREGE = self-reported external gain expectations; CI = confidence interval; LL = lower limit; UL = upper limit. Compound overreporting [(*z*SIMS + Fs T score) / 2] is the dependent variable. ^a^*p* <.001

## Discussion

Our study had two main findings. First, although our sample (hospital patients) differed considerably from the samples in the Brady et al. (2017) and Merckelbach et al. (2018) studies (PTSD veterans and forensic and non-forensic participants, respectively), we replicated their results, finding a significant positive association between alexithymia and symptom overreporting indices. In our study, the correlation of alexithymia with the SIMS (*r* = 0.45) was close to that reported by Brady et al. (2017) for the M-FAST (*r *= 0.49) and those reported by Merckelbach et al. (2018) for the SIMS (*r*s = 0.51–0.56). For the Infrequent somatic response scale (Fs) of the MMPI-2-RF used in our study, the correlation with alexithymia was lower but still significant (*r* = 0.26). It is worth noting that the proportion of patients exceeding the cutoff of 16 on the SIMS was substantially higher than that of patients scoring above 100 T on the MMPI-2-RF Fs scale (26.2% vs. 3.6%). This difference is likely due to the more stringent and somatic focus of the Fs, as well as the lower specificity of the SIMS at commonly used cutoffs (Shura et al., 2022; van Impelen et al., 2014; Wisdom et al., 2010). Nevertheless, both instruments showed consistent correlations with alexithymia, indicating that the association between alexithymia and symptom overreporting is robust across measures with different sensitivity profiles and not limited to specific populations. Furthermore, consistent with Brady et al. (2017) and Merckelbach et al. (2018), we found that symptom overreporting tendencies correlate most strongly with the DIF factor of the TAS-20, to a lesser extent with the DDF factor, and not with the EOT factor. This observation has clinical relevance, as several studies have noted that DIF in particular predicts unfavorable treatment outcomes—such as high levels of residual symptoms (Luminet et al., 2021; Pinna et al., 2020)—an issue we will revisit below.

Second, our analyses revealed no support for the notion that the relationship between alexithymia and symptom overreporting is merely a spurious artifact caused by individuals with external gain expectations exaggerating across all measures. The persistence of significant correlations between alexithymia and symptom overreporting among participants *without* self-reported external gain expectations counters this explanation. Notably, correlations in this group were not significantly weaker compared to those observed in the group with self-reported external gain expectations. Moreover, within the group without self-reported external gain expectations, there was no indication that the associations between alexithymia and symptom overreporting were driven solely by participants at the lower ends of the distributions: The proportions of individuals in this group exceeding the cut-off scores for both alexithymia and overreporting were similar to those in the group with reported external gain expectations.

One interesting, though difficult-to-explain, pattern in the current study was that participants with self-reported external gain expectations did not score above the cutpoints of the SIMS or Fs more often than those without such reported expectations. While we have no ready explanation for this finding, it underscores the complex relationship between incentives and symptom overreporting. These findings align with those of Dandachi-FitzGerald et al. (2016), who also found in their sample of hospital outpatients (*N* = 469) that external gain cannot fully explain symptom overreporting. Our regression analysis further demonstrated that alexithymia predicted symptom overreporting independently of self-reported external gain expectations.

More broadly, our findings support the view, recognized by experts in the field (e.g., Sweet et al., 2021), that symptom overreporting in clinical populations may be influenced by multiple factors, rather than solely by external incentives. Specifically, external incentives, such as financial or legal gains, often play a dominant role in forensic assessments (Bianchini et al., 2006; McDermott et al., 2013). However, our findings, alongside previous research (e.g., Brady et al., 2017; Merckelbach et al., 2019), underscore alexithymia as an additional and meaningful correlate of symptom overreporting, particularly in non-forensic populations. Importantly, alexithymia is relatively common, with prevalence estimates ranging from 7% to 13% in the general population to approximately 30% in clinical samples, and reaching up to 40% in specific patient groups such as individuals with major depression (Honkalampi et al., 2000; Luminet & Nielson, 2025). This is consistent with the prevalence observed in our own sample of hospital patients referred for psychological assessment (i.e., 32.1%), underscoring alexithymia as a clinically relevant factor to consider when interpreting symptom overreporting.

When patients are asked to report the frequency and/or intensity of their symptoms, they must perform a scaling (i.e., calibration) task. Inaccuracies or discrepancies in such reports do not necessarily indicate feigning (see Edwards et al., 2023, for a similar argument); rather, they may reflect patients’ intrinsic difficulties in evaluating and reporting internal sensations, a hallmark of alexithymia. Clinicians are therefore advised to consider both personality traits, such as alexithymia, and potential external incentives when assessing symptom overreporting. This approach can promote more accurate evaluations and guide tailored treatment strategies.

Building on these considerations, two key clinical implications emerge from the association between alexithymia and symptom overreporting. First, patients with high levels of alexithymia often present with comorbid conditions (e.g., Grabe et al., 2008; Palma-Álvarez et al., 2021). Given their difficulties in clearly articulating their symptoms (e.g., Luminet et al., 2021), elevated scores on symptom overreporting measures—such as the SIMS or the Fs scale of the MMPI-2-RF—should caution clinicians against settling prematurely on a single diagnostic category. We argue that the coexistence of high alexithymia and symptom overreporting signals diagnostic complexity. In particular, the DIF factor, combined symptom exaggeration, may hamper patients’ ability to communicate emotions and psychological concerns, leaving therapists with vague complaints and difficulties formulating effective treatment plans (Pinna et al., 2020). Clinicians may therefore benefit from incorporating additional information sources, such as collateral reports from family members or relevant records (e.g., school records).

Second, and perhaps more directly relevant to therapy, the combination of alexithymia and symptom exaggeration suggests that patients may benefit from interventions aimed at helping them identify and label emotions and experiences. Such interventions may help patients construct a clearer and more coherent narrative of their symptoms (e.g., Luminet & Nielson, 2025; Ogrodniczuk et al., 2011; Swiller, 1988).

Several limitations of the current study warrant commentary. First, our study relied on what patients themselves report regarding their external gain expectations. Our assumption, supported by prior work (Van Egmond & Kummeling, 2002), was that patients would be forthcoming about these expectations. However, they may not have fully disclosed, or been able to accurately convey, the entirety of their motivations (Chafetz et al., 2025; Merten, 2025). In future research, it would be worthwhile to ask family members about potential external gain interests that may be involved. Meanwhile, it should be noted that, as already mentioned, Dandachi-FitzGerald et al. (2016) had clinicians assess external gain expectations based on their interview and patient records. In that study, no relationship was found between external gain and symptom overreporting either. A related consideration is that our questionnaire focused on limited number of external gain domains, whereas a wider variety of gain expectations may contribute. Factors such as a desire for medical attention, wanting to be taken seriously by healthcare providers, or seeking to excuse a failure (Chafetz et al., 2020; Dandachi-FitzGerald et al., 2020b) could influence symptom overreporting and would be important to capture in future assessments.

A second limitation is that alexithymia scores were also based on self-report. The TAS-20 has a long and excellent track record (e.g., Bagby et al., 2021b; Luminet & Nielson, 2025). Still, it would be interesting to investigate whether symptom overreporting and alexithymia are related when alexithymia is measured using observer scales, such as the Toronto Structured Interview for Alexithymia (TSIA; Bagby et al., 2005) or the TAS-20 Informant Form (TAS-20-IF; Bagby et al., 2021a).

A third limitation is that our study was correlational in nature and does not allow for testing causal models. Future studies may want to track overreporting and alexithymia scores over a longer period, and longitudinal data collected in this way would allow for testing more refined causal models. Lastly, our sample was drawn from a single hospital in the Netherlands, which may limit the generalizability of our findings to other cultural or healthcare settings.

In conclusion, our study revealed that, in hospital patients referred for psychological issues, alexithymia is linked to symptom overreporting, and this relationship is independent of self-reported external gain expectations. This association may inform our understanding of symptom overreporting in clinical contexts. Symptom overreporting is often conceptualized as a red flag, signaling that all other psychological test data should be regarded as unreliable. In this context, McWhirter et al. (2020, p. 950) compare symptom overreporting to movement artefacts in MRI scans: “One analogy is of movement artefact on an MRI scan; there are many reasons a person might move during an MRI, but a single common end result: degradation of the images so that they are difficult or impossible to interpret.” While we do not dispute this metaphor, our findings suggest that symptom overreporting may involve more than a mere artefact. Its association with alexithymia points to a more fundamental difficulty in symptom calibration, one that provide a useful starting point for treatment.

## Supplementary Material

Accepted_Supplemental_Table_1_acag048

## Data Availability

The anonymized dataset supporting the findings of this study is accessible via Dataverse NL at https://doi.org/10.34894/VOL0PF. The analysis syntax is accessible via the Open Science Framework at: https://osf.io/g34y9.
