# Supplementary material for: Alexithymia, self-reported external gain expectations, and overreporting on symptom validity tests in hospital patients: further evidence for the relevance of alexithymia
Source: Arch Clin Neuropsychol. 2026 Jul 10;41(5):acag048. doi: 10.1093/arclin/acag048 (PMC13354059; doi:10.1093/arclin/acag048)
Supplement: Accepted_Supplemental_Table_1_acag048 [file accepted_supplemental_table_1_acag048.docx]

**Supplemental Table 1**

*Spearman rank correlations (and their 95% CI’s) between TAS, SIMS, and Fs of the MMPI-2-RF in patients with (n = 73) and without (n = 84) self-reported external gain expectations. Numbers (proportions) in both groups who scored above the cutpoint 19 of the SIMS and the cutpoint 80 of the Fs scale are also shown.*

|  | Self-reported  external gain expectations | |  |  |
| --- | --- | --- | --- | --- |
|  | Yes (*n* = 73) | No (*n* = 84) | *Z/ꭓ2* | *p* |
| *r*(TAS-20, SIMS) | .47 [.27, .64] | . 41 [.22, .58] | 0.53 | .300 |
| *r*(TAS-20, Fs) | .20 [-.04, .42] | .38 [.18, .56] | 1.21 | .113 |
| *n* (%) > cutpoint SIMS (19) | 6 (8.2%) | 18 (21.4%) | -2.29 | .022 |
| *n* (%) > cutpoint Fs (80) | 11 (15.1%) | 17 (20.2%) | -0.83 | .407 |

*Note*: TAS-20= 20-item Toronto Alexithymia Scale; SIMS=Structured Inventory of Malingered Symptomatology; Fs= Infrequent somatic responses scale of the Minnesota Multiphasic Personality Inventory–2 Restructured Form (MMPI-2-RF). ꭓ2 test statistics and corresponding *p*-values for comparisons involving numbers/proportions scoring above the SIMS or Fs cutpoints are also given.
